# Indole-3 Carbinol and Diindolylmethane Mitigated β-Amyloid-Induced Neurotoxicity and Acetylcholinesterase Enzyme Activity: In Silico, In Vitro, and Network Pharmacology Study

**DOI:** 10.3390/diseases12080184

**Published:** 2024-08-16

**Authors:** Kakarla Ramakrishna, Praditha Karuturi, Queen Siakabinga, Gajendra T.A., Sairam Krishnamurthy, Shreya Singh, Sonia Kumari, G. Siva Kumar, M. Elizabeth Sobhia, Sachchida Nand Rai

**Affiliations:** 1 KL College of Pharmacy, Koneru Lakshmaiah Education Foundation, Green Fields, Vaddeswaram, Guntur 522302, Andhra Pradesh, India; 2 Department of Pharmaceutical Engineering and Technology, Indian Institute of Technology, IIT BHU, Varanasi 221005, Uttar Pradesh, India; 3 SBS College of Pharmacy, Malwan, Fatehpur 212664, Uttar Pradesh, India; 4 Department of Pharmacoinformatics, National Institute of Pharmaceutical Education and Research (NIPER), Mohali 160062, Punjab, India; 5 Centre of Experimental Medicine and Surgery, Institute of Medical Sciences, Banaras Hindu University, Varanasi 221005, Uttar Pradesh, India

**Keywords:** indole-3 carbinol, diindolylmethane, Aβ toxicity, Alzheimer’s disease, acetylcholinesterase, neuroprotection, docking, molecular dynamics, network pharmacology

## Abstract

**Background:** Alzheimer’s disease (AD) is a neurodegenerative disease characterized by beta-amyloid (Aβ) deposition and increased acetylcholinesterase (AchE) enzyme activities. Indole 3 carbinol (I3C) and diindolylmethane (DIM) are reported to have neuroprotective activities against various neurological diseases, including ischemic stroke, Parkinson’s disease, neonatal asphyxia, depression, stress, neuroinflammation, and excitotoxicity, except for AD. In the present study, we have investigated the anti-AD effects of I3C and DIM. **Methods:** Docking and molecular dynamic studies against AchE enzyme and network pharmacological studies were conducted for I3C and DIM. I3C and DIM’s neuroprotective effects against self and AchE-induced Aβ aggregation were investigated. The neuroprotective effects of I3C and DIM against Aβ-induced neurotoxicity were assessed in SH-S5Y5 cells by observing cell viability and ROS. **Results:** Docking studies against AchE enzyme with I3C and DIM show binding efficiency of −7.0 and −10.3, respectively, and molecular dynamics studies revealed a better interaction and stability between I3C and AchE and DIM and AchE. Network pharmacological studies indicated that I3C and DIM interacted with several proteins involved in the pathophysiology of AD. Further, I3C and DIM significantly inhibited the AchE (IC_50_: I3C (18.98 µM) and DIM (11.84 µM)) and self-induced Aβ aggregation. Both compounds enhanced the viability of SH-S5Y5 cells that are exposed to Aβ and reduced ROS. Further, I3C and DIM show equipotential neuroprotection when compared to donepezil. **Conclusions:** Our findings indicate that both I3C and DIM show anti-AD effects by inhibiting the Aβ induced neurotoxicity and AchE activities.

## 1. Introduction

Accumulation of amyloid beta (Aβ) in neurons is the major cause of Alzheimer’s disease (AD), and increased acetylcholinesterase (AchE) activities in AD lead to memory impairment. To date, 60–70% of dementias are due to AD, and it is expected that at least 150 million people will be affected by AD by 2050 [1,2]. AD is associated with multifactorial abnormalities, including Aβ formation, aggregation, and accumulation in neurons, oxidative stress, and inflammation [3], which eventually lead to cognition and memory impairment. Acetylcholine (Ach) is the major neurotransmitter involved in the regulation of cognition and memory [4]. AchE is the key enzyme that maintains a sufficient amount of Ach levels in neurons. It has been reported that enhanced AchE activity is linked to promoting the accumulation of Aβ in AD [5]. Currently, n-methyl-D-aspartate (NMDA) antagonists and AchE inhibitors are used to improve cognitive and memory function in AD patients [6,7]. However, at present, a complete cure for AD is not available. Therefore, there has been a greater interest in finding novel molecules for the cure of AD [8].

Several medicinal plants and their phytochemical constituents have been identified as potent anti-AD compounds [9]. Indole-3-Carbinol (I3C) is one of the major phytochemicals present in cruciferous vegetables [10] and is reported to have antioxidant and anti-inflammatory activities that are responsible for cardioprotection, hepatoprotection, and neuroprotection [11]. I3C exhibited neuroprotection against multiple neurological diseases [12], including ischemic stroke [13,14,15], neonatal anoxia-induced neurodevelopmental deficits [16], prevented chronic social defeat stress (CSDS) [17], clonidine-mediated depression in rats [18], encephalopathy [19], and Parkinson’s disease (PD) [20,21]. Diindolylmethane (DIM) is the major metabolite (50%) or condensation product of I3C and is believed to mediate the pharmacological activity of I3C [22]. For instance, DIM exhibited a significant amelioration of platelet aggregation and thrombosis antioxidant mechanisms than its parent compound, I3C [23]. DIM offers neuroprotection in multiple diseases [12], including ischemic stroke [24,25], perinatal asphyxia [26], intracerebral hemorrhage (ICH) [27], multiple sclerosis [28], depression [29], and pain [30,31]. Cumulatively, I3C and DIM offer a wide range of protection against neurological diseases, such as stroke, PD, neonatal asphyxia, depression, stress, and drug-induced neurotoxicity [12]. However, the neuroprotective effects of I3C and DIM against Aβ induced neurotoxicity and AchE activity inhibition properties have not been studied. Therefore, in the present study, we investigated I3C and DIM neuroprotective effects against Aβ toxicity and AchE inhibiting activity using in silico, network pharmacology, and in vitro studies.

## 2. Materials and Methods

### 2.1. Chemicals

Indole-3 carbinol, diindolylmethane, AchE, MTT, and Aβ were obtained from Sigma Aldrich, India. All other chemicals used in the present study were obtained from high analytical grades from local suppliers. Neuroblastoma cells (SH-S5Y5 cells) were obtained for the National Centre of Cell Science (NCCS), Pune, India.

### 2.2. In Silico Docking

The binding affinity of I3C and DIM with AchE enzyme was investigated using docking studies. Briefly, the human AchE (4EY7) enzyme crystal structure was downloaded from the Protein Data Bank (PDB) from www.rcsb.org (https://www.rcsb.org/structure/4EY7, accessed on 24 March 2023). After that, the protein was prepared by removing the water, heteroatoms, and ligands using ADTtools (version 1.5.7) software. Similarly, the ligand was downloaded from Pubchem, and energy was minimized using chem 3D software (version 15.0). Both the prepared protein and ligand were converted to pdbqt format from pdb format. Then, the grid was generated such that it covered the complete AchE protein with a grid spacing of 0.90 Å, and the grid center was placed at coordinates 1.66, −47.349, and 28.143 (x, y, and z, respectively). Docking of I3C, DIM, and DPZ was performed with AutoDock 4.0 using the Lamarckian Genetic Algorithm (LGA). It applied a semi-empirical free energy force field to evaluate conformers generated by docking simulations. Furthermore, the discovery Studio 2016 was used for the visualization of the docked structures and binding interactions between lingands and AchE protein [32].

### 2.3. Molecular Dynamics

I3C, DIM, and DPZ are complexed to AchE (PDB ID: 4EY7) and were subjected to molecular simulation (MD) studies using Assisted Model Building with Energy Refinement software (AMBER 18) for 100 ns to study the dynamic nature of the complex. All the ligands were parameterized using the Antechamber module. For protein preparation, the tLEaP module of Amber was used. The ff14SB force field was used for the protein, and the GAFF force field was used for the small molecule. The tLEaP program adds the missing hydrogen atoms and assigns the molecular mechanics parameters. To build the system, the explicit solvent TIP3P water was used with an octahedral box of 10 Å. The charge on the complex was calculated, and obligatory Na^+^ counterions were added to neutralize the system to the required amount. The topology and parameter files were saved for each complex. After the preparation of the system, a sander module was used for the minimization. Two-step minimization was used; in the first minimization, only TIP3P waters were allowed to move, whereas in the second minimization, the whole system was allowed to move. Following minimization, the system was heated up from 0.0 K to 300.0 K using Langevin dynamics with a collisional frequency of 1 ps. For final production, the Langevin Thermostat was used for 100 ns, providing 100,000,000 steps with a time step of 1 fs, and after every 10 ps, coordinates were written to the trajectory file, yielding 1000 frames for each complex. After completion of the production run, the cpptraj module of amber and VMD was used for post-MD analysis.

### 2.4. Network Pharmacology Studies

We used network pharmacological approaches to predict the protein–protein interactions between I3C and AD and DIM and AD. Briefly, I3C, DIM, and AD genes were identified using the following keywords: indole 3 carbinol, diindolylmethane, and Alzheimer’s disease in the Genecards.org database. Then, the total number of genes was documented. Further, using the Venny diagram (Venny 2.1.0), the combined genes were identified between the I3C and AD and DIM and AD. **Protein–protein interaction (PPI):** The physical and functional interactions of overlapped genes were analyzed at a high confidence level of 0.9 by constructing the PPI using the Search Tool for the Retrieval of Interacting Genes/Protein (STRING). The protein (node) with no interactions (edges) was excluded, and the PPI was exported to the Cyoscape tool (Cytoscape version 3.10.1) to determine the topological feature of the network. Further, the top 10 target genes were enriched with strong PPI using the Cytoscape tool. **Gene Enrichment Analysis:** The PPI network constructed from the STRING was analyzed using ShinyGO 0.77 (http://bioinformatics.sdstate.edu/go/ (accessed on 29 November 2023)). The genomic information of PPI was retrieved in terms of biological processes, molecular functions, and cellular components with a gene enrichment set at *p*-value < 0.05, and the KEGG pathway was used to study the information related to retrieving the underlying metabolic pathways of targeted genes for Alzheimer’s disease. This *p*-value is set based on how likely it is that gene enrichment occurred by coincidence based on the false discovery rate (FDR).

### 2.5. In Vitro Studies

#### 2.5.1. Cholinesterase Inhibition Assay

Cholinesterase inhibitory activities of I3C and DIM were measured as reported earlier [33]. Briefly, I3C and DIM stock concentrations (1 mg/mL) were prepared in DMSO. Further, the stocking solution was prepared into six different working solutions (3.125, 6.25, 12.5, 25, 50, 100 µM). An equivalent concentration of I3C was taken to select the DIM concentration for the IC50 calculation. The reaction mixture consists of 100 µL of DTNB (0.0005 M), 50 µL of AchE (0.5 U mL^−1^), and 20 µL of ATCI as substrate (for AchE, 0.00375 M). I3C and DIM were preincubated for 10 min. Then, the AchE enzyme was added and incubated for 30 min. Thereafter, ATCI substrate was added to the above reaction mixture. DTNB’s interaction with thiocholines resulted in the formation of the yellow (5-thio-2-nitrobenzoate anion), which was observed for 20 min as a change in absorbance at 415 nm. Blank readings were taken with DMSO. Donepezil (DPZ) (0.001–50 µM) was used as a standard compound as an AchE inhibitor. The experiments were performed in triplicate (three separate runs), and the log concentration–percentage inhibition curves were used to graphically determine the IC_50_ values using GraphPad Prism 8.

#### 2.5.2. Amyloid β Inhibition Assay

**Preparation of Aβ_1–42_:** Aβ_1–42_ lyophilized peptide was obtained (Sigma Aldrich), and 1 mg of Aβ_1–42_ was dissolved in 80 µL ammonium hydroxide (NH_4_OH, 1%). To this, 920 µL of sterile phosphate buffer saline (PBS, pH 7.4) was added and stored at −80 °C until further analysis. The working solution of 10 µM of Aβ_1–42_ solution was prepared in PBS solution [34]. The Aβ_1–42_ inhibitory potential of I3C and DIM was assessed by measuring the Aβ_1–42_ self-induced aggregation and AchE-induced Aβ_1–42_ aggregation using thioflavin T assay. **Self-induced Aβ_1–42_ aggregation inhibition assay:** I3C and DIM-induced inhibitory effects on self-induced Aβ_1–42_ aggregation were analyzed as reported earlier [35]. Briefly, a reaction mixture containing 10 µL of Aβ_1–42_ (10 µM), PBS (50 µM) was incubated with 25, 50, and 100 µM of I3C and DIM for 48 h at 37 °C. After 48 h incubation, ThT (178 µL of 20 µM) was added, and fluorescence intensities were measured at 450 nm (excitation) and 485 nm (emission) [36]. **AchE induced Aβ_1–42_ aggregation inhibition assay:** 2 µL of Aβ_1–42_ (10 µM) and 16 µL of AchE (230 µM) was incubated with 25, 50, and 100 µM of I3C and DIM for 48 h at 37 °C. Following 48 h incubation, ThT (178 µL of 20 µM) was added, and fluorescence intensities were measured at 450 nm (excitation) and 485 nm (emission). In both the cases of self and AchE-induced Aβ_1–42_ aggregation inhibition assays, Aβ_1–42_ aggregation inhibition was reported as fluorescence intensities as normalized fluorescence intensities (NFI) with respect to normal control. DPZ (10 µL of 20 µM) was used as a reference compound [36].

### 2.6. Cell Culture Studies

The cell culture experiments were divided into two sub-sets of experiments. The first set of experiments involved the cytotoxic nature of I3C and DIM. I3C and DIM (25, 50, and 100 µM) were incubated with SH-S5Y5 cells (1 × 10^5^ cells/well) in 96 well plates for 24 h at 37 °C in a humidified chamber with 5% CO_2_ in an incubator. Then, MTT (20 µL) reagent was added to cells and allowed for 2 h. The formed, purple-colored formazan was dissolved in DMSO (100 µL), and the absorbance was measured at 520 nm. The second set of experiments investigated the neuroprotective effects of I3C and DIM against Aβ_1–42_-induced neurotoxicity in the SH-S5Y5 neuroblastoma cell line. Briefly, SH-S5Y5 cells (1 × 10^5^ cells/well) were grown for 24 h at 37 °C in a humidified chamber with 5% CO_2_ in an incubator. Then, Aβ_1–42_ (10 µM) was added and incubated for another 24 h. I3C, DIM (25, 50, and 100 µM), and DPZ (20 µM) were added to the cells and incubated for 72 h. Following 72 h incubation of I3C and DIM, MTT (20 µL) reagent was added to cells and allowed for 2 h. Then, the formed purple-colored formazan was dissolved in DMSO (100 µL), and the absorbance was measured at 520 nm [37]. The neuroprotective nature of I3C and DIM was counted based on their enhancement in the percentage of cell viability and reduction in reactive oxygen species (ROS) generation.

### 2.7. Measurement of Oxidative Stress

The intracellular reactive oxygen species (ROS) were detected using the 2,7-dichlorofluorescein diacetate (DCFDA). Briefly, SH-S5Y5 cells (1 × 10^5^ cells/well) after 72 h of treatment were incubated overnight with a freshly prepared solution of DCFDA (25 µM) for 45 min at 37 °C in the dark. Following incubation, cells were washed, and fluorescence signal was measured, 485 nm (excitation) and 535 nm (emission). Data were expressed as ROS levels as a percentage of control [38].

### 2.8. Statistical Analysis

All the data were analyzed using Graph Pad Prism 8. Data were represented as mean ± standard deviation. A *p*-value of less than 0.05 was considered statistically significant. The significant differences between groups were analyzed using one-way ANOVA.

## 3. Results

### 3.1. I3C and DIM Bind to AchE

**Figure 1:**
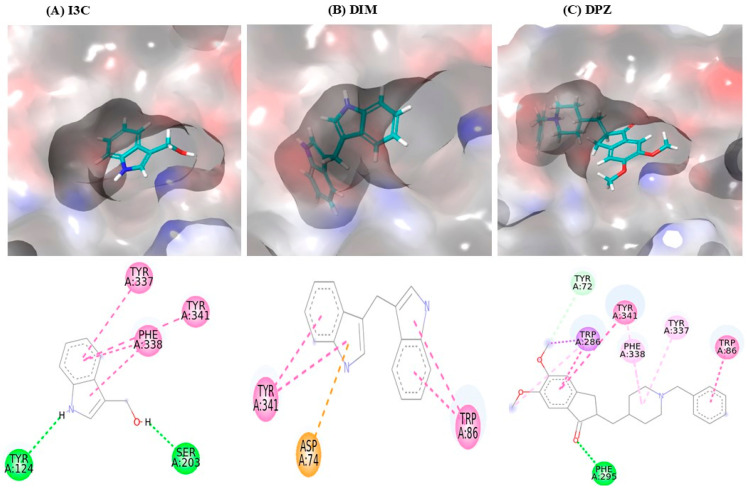
I3C and DIM binding interaction with the AchE. (**A**–**C**) represent the binding interactions (3D images) of I3C, DIM, and DPZ, respectively, with the AchE enzyme.

The interaction between I3C and DIM with AchE is depicted in Table 1, and the 3D interaction is depicted in Figure 1. I3C exhibited a binding affinity of −7.0 and interacted with AchE through various non-bonding interactions, including hydrogen bonds (Tyr124 and Ser203) and Pi-Pi stacking bonds (Tyr337, Phe338, and Tyr341). DIM had a higher binding affinity (−10.3) than I3C and exhibited multiple interactions with AchE, including Pi-Pi stacking bonds (Tyr86 and Tyr341) and Pi-anioinic interactions (Asp74). DPZ shows the highest binding affinity (−12.0) than I3C and DIM.

### 3.2. I3C and DIM Show High Stability with AchE

**Figure 2:**
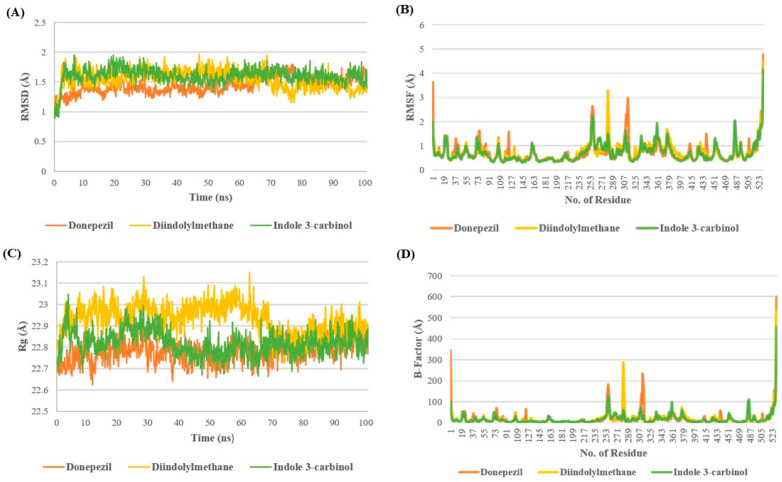
Molecular dynamics studies of I3C and DIM with AchE. (**A**–**D**) represent the RMSD, RMSF, Rg, and B-factor analysis for I3C, DIM, and DPZ with AchE.

The stability of the I3C and DIM complex with AchE is depicted in Figure 2. Root Mean square Deviation (RMSD) provides the deviation in the protein structure throughout the simulation by taking the minimization-2 topology file as a reference. The RMSD analysis revealed that all three complexes were stable at the end of the simulation with minor fluctuation of 1.5–1.7 Å. In the case of the DPZ complex, the RMSD remained below 1.5 Å till 70 ns; after 70ns, it increased >1.5 Å up to 1.7 Å, whereas in the case of DIM discerning the opposite pattern after 70 ns, RMSD decreased, showing the RMSD of 1.2–1.4 Å. However, the I3C complex showed minor fluctuations throughout the simulation after 8ns (Figure 2A).

The analysis of RMSF and B-factors offers insights into protein fluctuations during MD simulations. In the AchE complex with DPZ, higher fluctuations were observed compared with complexes with DIM and I3C. Specifically, DIM exhibited greater fluctuation in the 270–290 region, while DPZ showed increased fluctuations around 305–325 compared with I3C. Additionally, all three complexes exhibited minimal fluctuations around the binding site cavity (residues 72–86, 286–295, and 337–341), indicating restricted movement of binding site residues due to tight ligand binding (Figure 2B,D).

The radius of gyration (Rg) serves as a measure of the compactness of a complex throughout a simulation, reflecting its stability. Higher Rg values suggest lower compactness and potentially less stability, while lower values indicate greater compactness and stability. Remarkably, up to 70 ns, the DNZ complex exhibited a higher Rg value, indicating lower compactness, in comparison to the I3C and DIM complexes. However, following the initial 70 ns, all three complexes demonstrated comparable Rg values until the end of the simulation. This observation suggests that while the DNZ complex initially displayed less compactness, the complexes converged to similar levels of compactness throughout the simulation, possibly indicating equilibration or stabilization of the complexes’ structures (Figure 2C).

### 3.3. The Common Genes of I3C and DIM with AD

**Figure 3:**
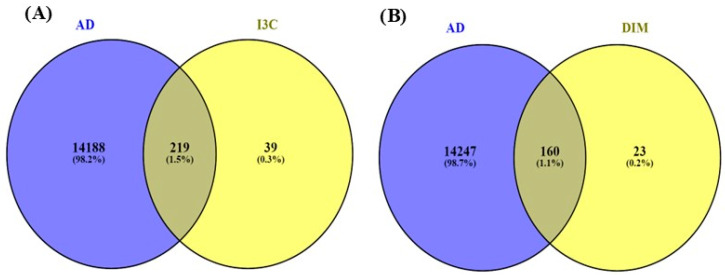
Venny diagram. (**A**,**B**) represent the common genes between I3C and DIM with AD. The intersection represents the overlapping genes.

Figure 3 indicates the overlapping genes of I3C, DIM, and AD. The overall genes for I3C, DIM, and AD were found with the help of Genecards.com. Then, using the Venny diagram (Venny version 2.1.0), we found overlapping genes for both the I3C and DIM with AD. Further, 219 overlapping genes are commonly found in I3C and AD, and 160 genes are commonly found in DIM and AD.

### 3.4. Protein–Protein Interaction Analysis

**Figure 4:**
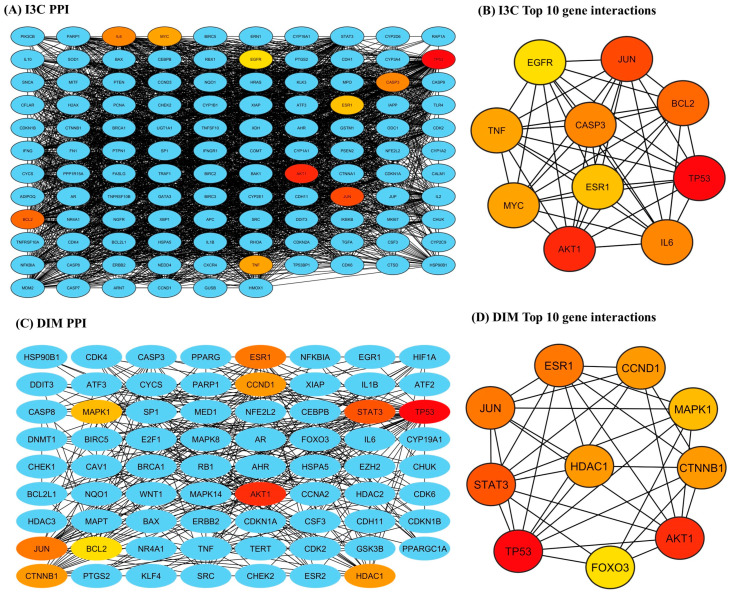
The PPI networks of I3C and DIM against the AD. (**A**,**C**) represent the PPI networks of I3C and DIM, respectively. (**B**,**D**) represent the top 10 genes that are associated with I3C and DIM against AD, respectively.

The STRING-based PPI interactions of I3C and DIM with AD are illustrated in Figure 4. The I3C PPI network has 116 nodes (proteins) connected by 2434 edges (interaction), and the DIM PPI network has 71 nodes with 350 edges (interactions), with a local clustering coefficient of 0.724 (I3C) and 0.530 (DIM) (Figure 4A and 4C respectively). We found the *p*-value (PPI enrichment) was less than 1, indicating that the acquired network significantly produced more interactions than expected. We found that at a 0.9 confidence level, multiple target genes were excluded from STRING and the central hub due to zero-degree node interactions. Further, the top 10 target genes for I3C based on the degree of topological analysis were found to be as follows: TP53 (107), AKT 1 (98), JUN (95), BCL2 (91), CASP3 (89), IL6 (89), MYC (86), TNF (86), ESR1 (85), and STAT3 (83) (Figure 4B). Similarly, the top 10 target genes for DIM were as follows: TP53 (46), AKT 1 (27), STAT3 (26), ESR1 (25), CTNNB1 (19), HDAC1 (19), CCND1 (19), MAPK1 (18), and FOXO3 (17) (Figure 4D).

### 3.5. Gene Enrichment Analysis

**Figure 5:**
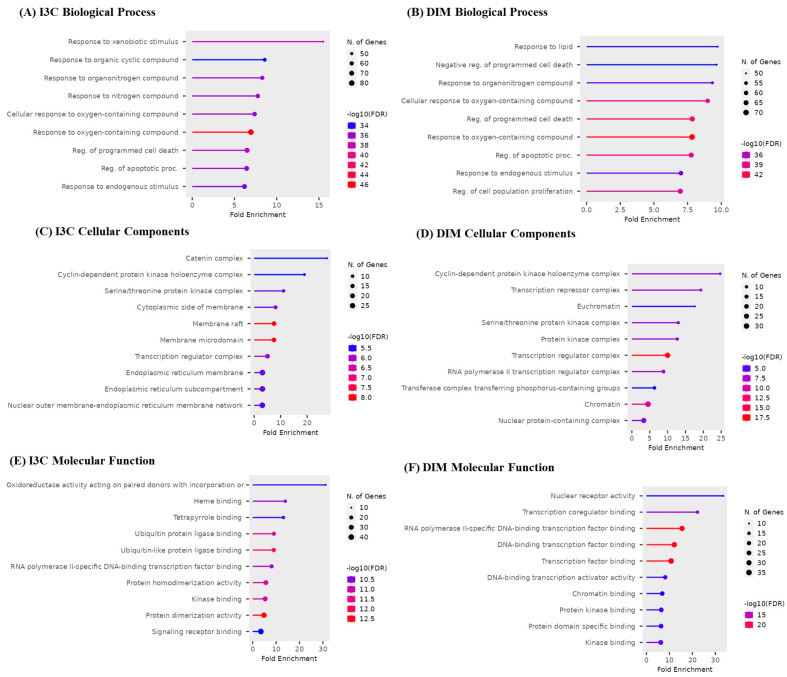
Gene enrichment analysis of I3C and DIM. (**A**,**B**) represent the biological process of I3C and DIM, respectively. (**C**,**D**) represent the cellular components of I3C and DIM, respectively. (**E**,**F**) represent the molecular function of I3C and DIM, respectively.

The top 10 GO biological, cellular, and molecular targets for I3C and DIM against AD are illustrated in Figure 5. I3C and DIM are involved in biological processes such as apoptosis, xenobiotic reactions, and cell proliferation (Figure 5A,B). Cellular components show that I3C and DIM are involved in several cellular activities, majorly endoplasmic reticulum, protein kinase, and nuclear functions (Figure 5C,D). Further, molecular processes show multiple functions related to DNA and protein and are responsible for kinase activity (Figure 5E,F).

### 3.6. I3C and DIM Inhibited Choline esterase Enzyme Activity

**Figure 6:**
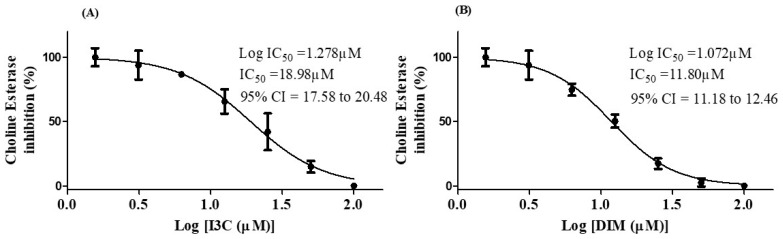
IC_50_ concentrations of I3C and DIM. (**A**,**B**) represent the IC_50_ values of I3C and DIM, respectively. The selected concentrations of I3C and DIM were run in triplicates.

The choline esterase inhibition potential of I3C and DIM is depicted in Figure 6. The IC_50_ values of I3C and DIM were found to be 18.98 µM and 11.84 µM, respectively. These results imply that DIM is a more potent inhibitor of choline esterase than I3C.

### 3.7. I3C and DIM Inhibited Aβ Aggregation

**Figure 7:**
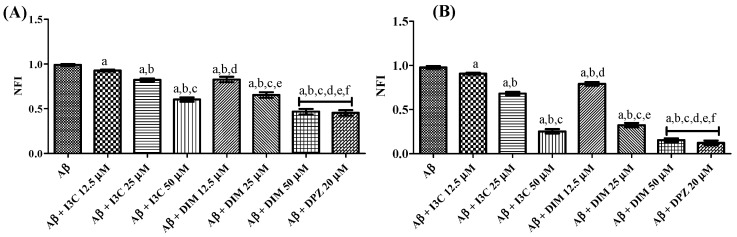
The effect of I3C and DIM on self-induced and AchE-induced Aβ aggregation. (**A**,**B**) represent self-induced Aβ aggregation and AchE-induced Aβ aggregation, respectively. ^a^ *p* < 0.001 vs. Aβ, ^b^ *p* < 0.001 I3C 12.5 µM, ^c^ *p* < 0.001 vs. I3C 25 µM, ^d^ *p* < 0.001 vs. I3C 50 µM, ^e^ *p* < 0.001 DIM vs. 12.5 µM, and ^f^ *p* < 0.001 vs. DIM 25 µM, respectively. All the experiments were run in run triplicates, and data are expressed in mean ± SD. One-way ANOVA followed by Tukey’s post hoc test.

The effect of I3C and DIM on self-induced and AchE-induced Aβ aggregation is illustrated in Figure 7. One ANOVA followed by Tukey’s post hoc analysis revealed a significant difference among groups. I3C incubation with Aβ dose-dependently reduced the Aβ self-induced aggregation (*p* < 0.001). Similarly, DIM also reduced the self-induced Aβ aggregation in a dose-dependent manner (*p* < 0.0001). There were no significant differences between DIM (50 µM) and DPZ (20 µM) (*p* > 0.05). However, no dose of I3C exhibits equipotent inhibition of self-induced Aβ aggregation compared with DPZ treatment. Identical results were obtained with the treatment of I3C and DIM against AchE-induced Aβ aggregation when compared to DPZ. Moreover, DIM was found to be a more potent inhibitor of self-induced and AchE Aβ aggregation than I3C (*p* < 0.001). These findings imply that both I3C and DIM show inhibition of Aβ aggregation.

### 3.8. I3C and DIM Mitigated Aβ Induced Neurotoxicity in SH-S5Y5 Cells

**Figure 8:**
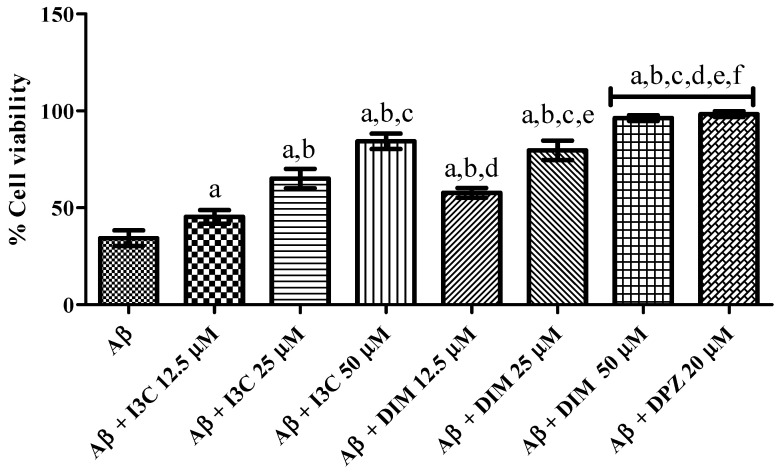
The effect of I3C and DIM treatment on cell viability is caused by Aβ-induced neurotoxicity. ^a^ *p* < 0.001 vs. Aβ, ^b^ *p* < 0.001 I3C 12.5 µM, ^c^ *p* < 0.001 vs. I3C 25 µM, ^d^ *p* < 0.001 vs. I3C 50 µM, ^e^ *p* < 0.001 DIM vs. 12.5 µM, and ^f^ *p* < 0.001 vs. DIM 25 µM, respectively. All the experiments were run in run triplicates, and data are expressed in Mean ± SD. One-way ANOVA followed by Tukey’s post hoc test.

The effect of I3C and DIM on Aβ induced neurotoxicity in SH-S5Y5 cells is illustrated in Figure 8. I3C treatment significantly ameliorated the Aβ induced neurotoxicity in terms of increased cell viability (%) in a dose-dependent manner (*p* < 0.001). We also observed significant improvement in cell viability (%) treatment with DIM (*p* < 0.0001). However, I3C (50 µM) treatment exhibited a lower percentage of cell viability than DIM (50 µM), indicating that DIM is a more potent compound to ameliorate Aβ induced neurotoxicity than I3C. Further, DIM, but not I3C, exhibited similar cell viability (%) when compared to DPZ.

### 3.9. I3C and DIM Attenuated Oxidative Stress

**Figure 9:**
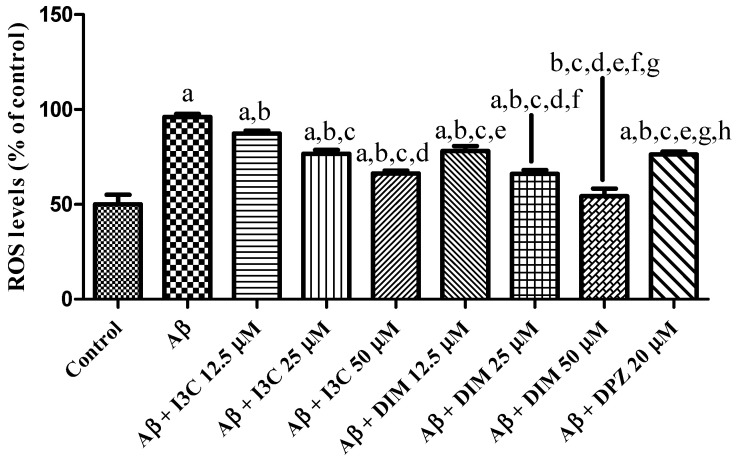
The effect of I3C and DIM treatment on Aβ-induced ROS levels. ^a^ *p* < 0.001 vs. Aβ, ^b^ *p* < 0.001 I3C 12.5 µM, ^c^ *p* < 0.001 vs. I3C 25 µM, ^d^ *p* < 0.001 vs. I3C 50 µM, ^e^ *p* < 0.001 I3C 50 µM, and ^f^ *p* < 0.001 vs. DIM 12.5 µM, ^g^ *p* < 0.001 vs. DIM 25 µM, and ^h^ *p* < 0.001 vs. DIM 50 µM, respectively. All the experiments were run in run triplicates, and data are expressed in mean ± SD. One-way ANOVA followed by Tukey’s post hoc test.

The effect of I3C and DIM on an induced increase in ROS levels is depicted in Figure 9. I3C treatment reduced the ROS levels in a dose-dependent manner (*p* < 0.001). Similarly, DIM treatment significantly reduced the Aβ induced ROS levels in a dose-dependent manner (*p* < 0.001). Further, I3C (50 µM) treatment shows less inhibition of ROS than DIM (50 µM), implying that DIM is a more potent antioxidant than I3C.

## 4. Discussion

AD is a complex disease, and to date, there is no therapy available to mitigate AD [9]. In this investigation, we report the anti-AD potential of two phytochemicals (I3C and DIM) from cruciferous vegetables. We found that I3C and DIM bind to the AchE enzyme and inhibit its activity, implying that both the compounds can be considered as AchE inhibitors. Moreover, our findings demonstrate that I3C and DIM mitigated the Aβ-induced neurotoxicity, thereby exerting anti-AD effects.

Acetylcholine esterase (AchE) is a critical enzyme that is responsible for the degradation of acetylcholine (Ach), a neurotransmitter responsible for cognition and memory function [8,39]. It has been observed that Ach levels drastically decreased in AD patients due to increased AchE enzyme activity, leading to cognition and memory impairment [7]. Moreover, increased AchE activity is positively correlated with the augmentation of Aβ plaques in AD [40]. As of now, AchE inhibitors are approved by the FDA and are currently used in clinics for the symptomatic management of AD. Therefore, the inhibition of AchE is considered a potential therapeutic strategy for mitigating the cognitive and memory impairment associated with AD. In view of this, we have investigated the I3C and DIM binding and inhibitory properties of AchE and its mediated Aβ-induced neurotoxicity.

Earlier findings report that I3C and DIM offer neuroprotection in several neurological diseases [12,15]. However, there is no evidence of I3C and DIM-mediated neuroprotection role in AD. Our in silico studies show that I3C and DIM show multiple interactions with AchE enzyme, including hydrogen bonds, Pi-Pi stacking, and Pi-anion interactions with overall binding efficiency of −7.0 and −10.3, respectively, for I3C and DIM. Further, we found that the DIM binding affinity (−10.3) is near to the binding efficiency of DPZ (−12.0). These results conclude that both I3C and DIM exhibit AchE binding properties. Moreover, we observed that DIM has a higher binding affinity (−10.3) to the AchE enzyme than its parent compound I3C (−7.0). Correspondingly, molecular dynamics studies showed that DIM attained the highest stability after 70 ns, with an average RMSD value of 1.41 Å compared with DPZ (1.57 Å) and I3C (1.59 Å). Additionally, RMSD analysis indicated that DIM displayed greater stability compared with both DPZ and its parent molecule, I3C. Likewise, the radius of gyration after 70 ns, DIM exhibited nearly identical values to DPZ and I3C, averaging 22.8 Å. Overall, the MD studies consistently reported that DIM possesses greater stability compared with DPZ and I3C, suggesting a higher affinity for binding to the AchE enzyme.

We found the IC_50_ value of 18.98 µM for I3C and 11.80 µM for DIM against AchE enzyme inhibition, suggesting that DIM shows significant inhibition of choline esterase than I3C. Based on the above findings, we further investigated the Aβ aggregation inhibitory potential of I3C and DIM. We found significant inhibition of Aβ-self-induced aggregation and AchE-induced Aβ aggregation with the incubation of I3C and DIM. Among all I3C doses, 50 µM dose of I3C shows a better inhibition of self and AchE-induced Aβ aggregation but shows less Aβ aggregation inhibition when compared with donepezil (DPZ). Oppositely, DIM (50 µM dose) shows equipotential activity with DPZ (10 µM), suggesting that DIM is a potential anti-Aβ aggregating agent than I3C. Correspondingly, we also found that DIM shows better inhibition of Aβ aggregation induced by AchE than I3C. Similarly, DIM shows equipotential inhibition of AchE-induced Aβ aggregation when compared to DPZ. Hence, these findings emphasize that I3C and DIM can inhibit the β-amyloid aggregation. Several reports suggest that the Aβ accumulation in neurons enhances neuronal cell death [33,37]. Our results show that I3C and DIM pretreatment increased the cell viability of SH-S5Y5 neuronal cells exposed to Aβ. Further, I3C and DIM also reduced the reactive oxygen species (ROS) in SH-S5Y5 neuronal cells incubated Aβ, indicating that the antioxidant properties of I3C and DIM might be another reason for increased cell viability after Aβ exposure. Indeed, this study aimed to evaluate the neuroprotective effects of I3C and DIM against AD. Surprisingly, we found better anti-AD effects with DIM with its parent compound, I3C. Previous findings suggested that DIM mediates the pharmacological actions of I3C [22]. In supporting these observations, our study also shows significant neuroprotection with DIM treatment against AD than I3C, indicating that I3C protective effects are partially mediated by DIM.

Network pharmacological approaches are widely used to find out the proteins or genes involved in the protective response of drugs or novel chemical entities against selected diseases. This approach allows the researchers to predict the genes or proteins involved in the mechanism of action of drugs. Here, we applied the network pharmacological approaches to identify the genes/proteins that are involved in the mechanism of action of I3C and DIM against AD. We constructed a PPI network and found that 116 and 71 proteins for I3C and DIM, respectively, interacted with each other. Then we identified the top 10 genes for I3C, including TP53, AKT 1, JUN, BCL2, CASP3, IL6, MYC, TNF, ESR1, and STAT3. Similarly, the top 10 target genes for DIM included TP53, AKT 1, STAT3, ESR1, CTNNB1, HDAC1, CCND1, MAPK1, and FOXO3. These proteins are involved in the cell division, cell growth (TP53, AKT1, MYC, CCND1, HDAC1, and MAPK1), inflammation (JUN, IL6, TNF, and STAT3), apoptosis (BCL2 and CASP3), cell regeneration and proliferation (CTNNB1), and autophagy, cell proliferation, and cell survival (FOXO3). Indeed, most of these proteins have been linked to the progress of AD. Therefore, our findings suggest that I3C and DIM would interact with many of the pathological events that contribute to AD pathogenesis. Further, we analyzed the AD KEGG pathway using ShinyGO, suggesting that multiple genes are involved in the MOA of I3C and DIM (highlighted in red, Supplementary Figures S1 and S2). The probable molecular pathway underlying a therapeutic intervention can be mapped out or traced with a network pharmacology approach. However, to ensure the reliability of anticipated interactions and their implications for AD, experimental validation via in vitro and in-vivo studies should be performed and evaluated.

In this preliminary study, we showed AchE enzyme and Aβ inhibition with the intervention of I3C and DIM. Evidence from earlier reports shows inhibition of inflammation and apoptosis with the treatment of I3C and DIM in other neurological diseases. Further, we did not investigate the anti-inflammatory and antiapoptotic effects of I3C and DIM against AD. Therefore, future studies need to investigate the above-mentioned anti-AD effects of I3C and DIM against in vivo studies.

## 5. Conclusions

In conclusion, the present study reported the anti-Alzheimer’s effects of I3C and DIM through the inhibition of the AchE and Aβ aggregation. In silico studies confirm that I3C and DIM interact with the AchE enzyme and show a potent binding efficiency. Further, network pharmacological assays show that I3C and DIM interacted with several proteins associated with AD pathogenesis. Cumulatively, our investigation suggests that I3C and DIM show anti-AD effects.

## Figures and Tables

**Table 1:** I3C and DIM binding affinities with AchE.

Sr. No.	Ligands Name	Binding Affinity	Interactions				
			Hydrogen Bonds	Pi-Pi Stacking	Pi-Alkyl	Pi-Sigma	Pi-Anion
1	Indole 3-Carbinol	−7.0	Tyr124, Ser203	Tyr337, Phe338, Tyr341	-	-	-
2	Diindolylmethane	−10.3	-	Tyr341, Trp86	-	-	Asp74
3	Donepezil (DPZ)	−12.0	Tyr72, Phe295	Tyr341, Trp286, Trp86	Trp286, Tyr341, Phe338, Tyr337	Trp286	-

I3C and DIM interacted with the AchE enzyme through various interactions, including hydrogen bonding, Pi-Pi Stacking, Pi-Alkyl, Pi-Sigma, and Pi-anion bonding.

## Data Availability

Data are contained within the article and Supplementary Materials.

## Supplementary Materials

The following supporting information can be downloaded at https://www.mdpi.com/article/10.3390/diseases12080184/s1, Figure S1: KEGG analysis of I3C; Figure S2: KEGG analysis of DIM.

## References

[1] Nichols E., Szoeke C.E., Vollset S.E., Abbasi N., Abd-Allah F., Abdela J., Murray C.J. (2019). Global, regional, and national burden of Alzheimer’s disease and other dementias, 1990–2016: A systematic analysis for the Global Burden of Disease Study 2016. Lancet Neurol..

[2] Li X., Feng X., Sun X., Hou N., Han F., Liu Y. (2022). Global, regional, and national burden of Alzheimer’s disease and other dementias, 1990–2019. Front. Aging Neurosci..

[3] Kumar A., Singh A. (2015). A review on Alzheimer’s disease pathophysiology and its management: An update. Pharmacol. Rep..

[4] Martorana A., Esposito Z., Koch G. (2010). Beyond the cholinergic hypothesis: Do current drugs work in Alzheimer’s disease?. CNS Neurosci. Ther..

[5] Bartolini M., Bertucci C., Cavrini V., Andrisano V. (2003). β-Amyloid aggregation induced by human acetylcholinesterase: Inhibition studies. Biochem. Pharmacol..

[6] Pardo-Moreno T., González-Acedo A., Rivas-Domínguez A., García-Morales V., García-Cozar F.J., Ramos-Rodríguez J.J., Melguizo-Rodríguez L. (2022). Therapeutic approach to Alzheimer’s disease: Current treatments and new perspectives. Pharmaceutics.

[7] Tripathi P.N., Srivastava P., Sharma P., Tripathi M.K., Seth A., Tripathi A., Shrivastava S.K. (2019). Biphenyl-3-oxo-1,2,4-triazine linked piperazine derivatives as potential cholinesterase inhibitors with antioxidant property to improve the learning and memory. Bioorg. Chem..

[8] Rai S.N., Singh C., Singh A., Singh M.P., Singh B.K. (2020). Mitochondrial Dysfunction: A Potential Therapeutic Target to Treat Alzheimer’s Disease. Mol. Neurobiol..

[9] Tripathi P.N., Lodhi A., Rai S.N., Nandi N.K., Dumoga S., Yadav P., Chaudhary S. (2024). Review of Pharmacotherapeutic Targets in Alzheimer’s Disease and Its Management Using Traditional Medicinal Plants. Degener. Neurol. Neuromuscul. Dis..

[10] Aggarwal B.B., Ichikawa H. (2005). Molecular targets and anticancer potential of indole-3-carbinol and its derivatives. Cell Cycle.

[11] Singh A.A., Patil M.P., Kang M.J., Niyonizigiye I., Kim G.D. (2021). Biomedical application of Indole-3-carbinol: A mini-review. Phytochem. Lett..

[12] Singh A.A., Yadav D., Khan F., Song M. (2024). Indole-3-Carbinol and Its Derivatives as Neuroprotective Modulators. Brain Sci..

[13] Ramakrishna K., Jain S.K., Krishnamurthy S. (2022). Pharmacokinet. Pharmacodyn. Prop. Indole-3-Carbinol Exp. Focal Ischemic Injury. Eur. J. Drug Metab. Pharmacokinet..

[14] Paliwal P., Chauhan G., Gautam D., Dash D., Patne S.C., Krishnamurthy S. (2018). Indole-3-carbinol improves neurobehavioral symptoms in a cerebral ischemic stroke model. Naunyn-Schmiedeberg’s Arch. Pharmacol..

[15] Kakarla R., Karuturi P., Siakabinga Q., Kasi Viswanath M., Dumala N., Guntupalli C., Gujjari L. (2024). Current understanding and future directions of cruciferous vegetables and their phytochemicals to combat neurological diseases. Phytother. Res..

[16] Ramakrishna K., Krishnamurthy S. (2023). Indole-3-carbinol ameliorated the neurodevelopmental deficits in neonatal anoxic injury in rats. Int. J. Dev. Neurosci..

[17] Pan S., Ma Y., Yang R., Lu X., You Q., Ye T., Huang C. (2022). Indole-3-carbinol selectively prevents chronic stress-induced depression-but not anxiety-like behaviors via suppressing pro-inflammatory cytokine production and oxido-nitrosative stress in the brain. Front. Pharmacol..

[18] El-Naga R.N., Ahmed H.I., Al Haleem E.N.A. (2014). Effects of indole-3-carbinol on clonidine-induced neurotoxicity in rats: Impact on oxidative stress, inflammation, apoptosis and monoamine levels. Neurotoxicology.

[19] Ramakrishna K., Sinku S., Majumdar S., Singh N., Gajendra T.A., Rani A., Krishnamurthy S. (2023). Indole-3-carbinol ameliorated the thioacetamide-induced hepatic encephalopathy in rats. Toxicology.

[20] Mohamad K.A., El-Naga R.N., Wahdan S.A. (2022). Neuroprotective effects of indole-3-carbinol on the rotenone rat model of Parkinson’s disease: Impact of the SIRT1-AMPK signaling pathway. Toxicol. Appl. Pharmacol..

[21] Saini N., Akhtar A., Chauhan M., Dhingra N., Sah S.P. (2020). Protective effect of Indole-3-carbinol, an NF-κB inhibitor in experimental paradigm of Parkinson’s disease: In silico and in vivo studies. Brain Behav. Immun..

[22] Bradlow H., Zeligs M. (2010). Diindolylmethane (DIM) spontaneously forms from indole-3-carbinol (I3C) during cell culture experiments. In Vivo.

[23] Ramakrishna K., Singh N., Krishnamurthy S. (2022). Diindolylmethane ameliorates platelet aggregation and thrombosis: In silico, in vitro, and in vivo studies. Eur. J. Pharmacol..

[24] Dhir N., Jain A., Sharma A.R., Prakash A., Bhatia A., Medhi B. (2022). Neuroprotective effect of 3,3′-diindolylmethane and ɑ-naphthoflavone, aryl hydrocarbon receptor modulators in an experimental model of ischemic stroke. CNS Neurol. Disord. Drug Targets.

[25] Ramakrishna K., Singh S.K., Krishnamurthy S. (2022). Diindolylmethane Ameliorates Ischemic Stroke-Induced Brain Injury by Peripheral and Central Mechanisms. Curr. Neurovascular Res..

[26] Rzemieniec J., Bratek E., Wnuk A., Przepiórska K., Salińska E., Kajta M. (2020). Neuroprotective effect of 3,3′-Diindolylmethane against perinatal asphyxia involves inhibition of the AhR and NMDA signaling and hypermethylation of specific genes. Apoptosis.

[27] Matsumoto K., Kinoshita K., Yoshimizu A., Kurauchi Y., Hisatsune A., Seki T., Katsuki H. (2020). Laquinimod and 3,3′-diindolylemethane alleviate neuropathological events and neurological deficits in a mouse model of intracerebral hemorrhage. J. Neuroimmunol..

[28] Yang S., Tan L., Chen Y., Liu A., Hong M., Peng Z. (2020). DIM mitigates the development of experimental autoimmune encephalomyelitis by maintaining the stability and suppressive function of regulatory T cells. Cell. Immunol..

[29] Madison C.A., Kuempel J., Albrecht G.L., Hillbrick L., Jayaraman A., Safe S., Eitan S. (2022). 3,3′-Diindolylmethane and 1,4-dihydroxy-2-naphthoic acid prevent chronic mild stress induced depressive-like behaviors in female mice. J. Affect. Disord..

[30] Mattiazzi J., Sari MH M., de Bastos Brum T., Araújo PC O., Nadal J.M., Farago P.V., Cruz L. (2019). 3,3′-Diindolylmethane nanoencapsulation improves its antinociceptive action: Physicochemical and behavioral studies. Colloids Surf. B Biointerfaces.

[31] Mattiazzi J., Sari M.H.M., Araujo P.C.O., Englert A.V., Nadal J.M., Farago P.V., Cruz L. (2020). Ethylcellulose microparticles enhance 3,3′-diindolylmethane anti-hypernociceptive action in an animal model of acute inflammatory pain. J. Microencapsul..

[32] Morris G.M., Goodsell D.S., Halliday R.S., Huey R., Hart W.E., Belew R.K., Olson A.J. (1998). Automated docking using a Lamarckian genetic algorithm and an empirical binding free energy function. J. Comput. Chem..

[33] Gutti G., Leifeld J., Kakarla R., Bajad N.G., Ganeshpurkar A., Kumar A., Singh S.K. (2023). Discovery of triazole-bridged aryl adamantane analogs as an intriguing class of multifunctional agents for treatment of Alzheimer’s disease. Eur. J. Med. Chem..

[34] Gutti G., Kumar D., Paliwal P., Ganeshpurkar A., Lahre K., Kumar A., Singh S.K. (2019). Development of pyrazole and spiropyrazoline analogs as multifunctional agents for treatment of Alzheimer’s disease. Bioorg. Chem..

[35] Bhanukiran K., Singh R., Gajendra T.A., Ramakrishna K., Singh S.K., Krishnamurthy S., Hemalatha S. (2023). Vasicinone, a pyrroloquinazoline alkaloid from Adhatoda vasica Nees enhances memory and cognition by inhibiting cholinesterases in Alzheimer’s disease. Phytomed. Plus.

[36] Kumar D., Gupta S.K., Ganeshpurkar A., Gutti G., Krishnamurthy S., Modi G., Singh S.K. (2018). Development of Piperazinediones as dual inhibitor for treatment of Alzheimer’s disease. Eur. J. Med. Chem..

[37] Gutti G., Kakarla R., Kumar D., Beohar M., Ganeshpurkar A., Kumar A., Singh S.K. (2019). Discovery of novel series of 2-substituted benzo[d]oxazol-5-amine derivatives as multi-target directed ligands for the treatment of Alzheimer’s disease. Eur. J. Med. Chem..

[38] Mhillaj E., Papi M., Paciello F., Silvestrini A., Rolesi R., Palmieri V., Mancuso C. (2020). Celecoxib Exerts Neuroprotective Effects in β-Amyloid-Treated SH-SY5Y Cells through the Regulation of Heme Oxygenase-1: Novel Insights for an Old Drug. Front. Cell Dev. Biol..

[39] Singh M., Agarwal V., Pancham P., Jindal D., Agarwal S., Rai S.N., Gupta V. (2024). A Comprehensive Review and Androgen Deprivation Therapy and Its Impact on Alzheimer’s Disease Risk in Older Men with Prostate Cancer. Degener. Neurol. Neuromuscul. Dis..

[40] Srivastava P., Tripathi P.N., Sharma P., Rai S.N., Singh S.P., Srivastava R.K., Shrivastava S.K. (2019). Design and development of some phenyl benzoxazole derivatives as a potent acetylcholinesterase inhibitor with antioxidant property to enhance learning and memory. Eur. J. Med. Chem..

