# Supplementary material for: Indole-3 Carbinol and Diindolylmethane Mitigated β-Amyloid-Induced Neurotoxicity and Acetylcholinesterase Enzyme Activity: In Silico, In Vitro, and Network Pharmacology Study
Source: Diseases. 2024 Aug 16;12(8):184. doi: 10.3390/diseases12080184 (PMC11354007; doi:10.3390/diseases12080184)
Supplement: Supplementary file 1 [file diseases-12-00184-s001.zip › diseases-3136509-supplementary.pdf]

Supplementary figure S1: KEGG analysis of I3C

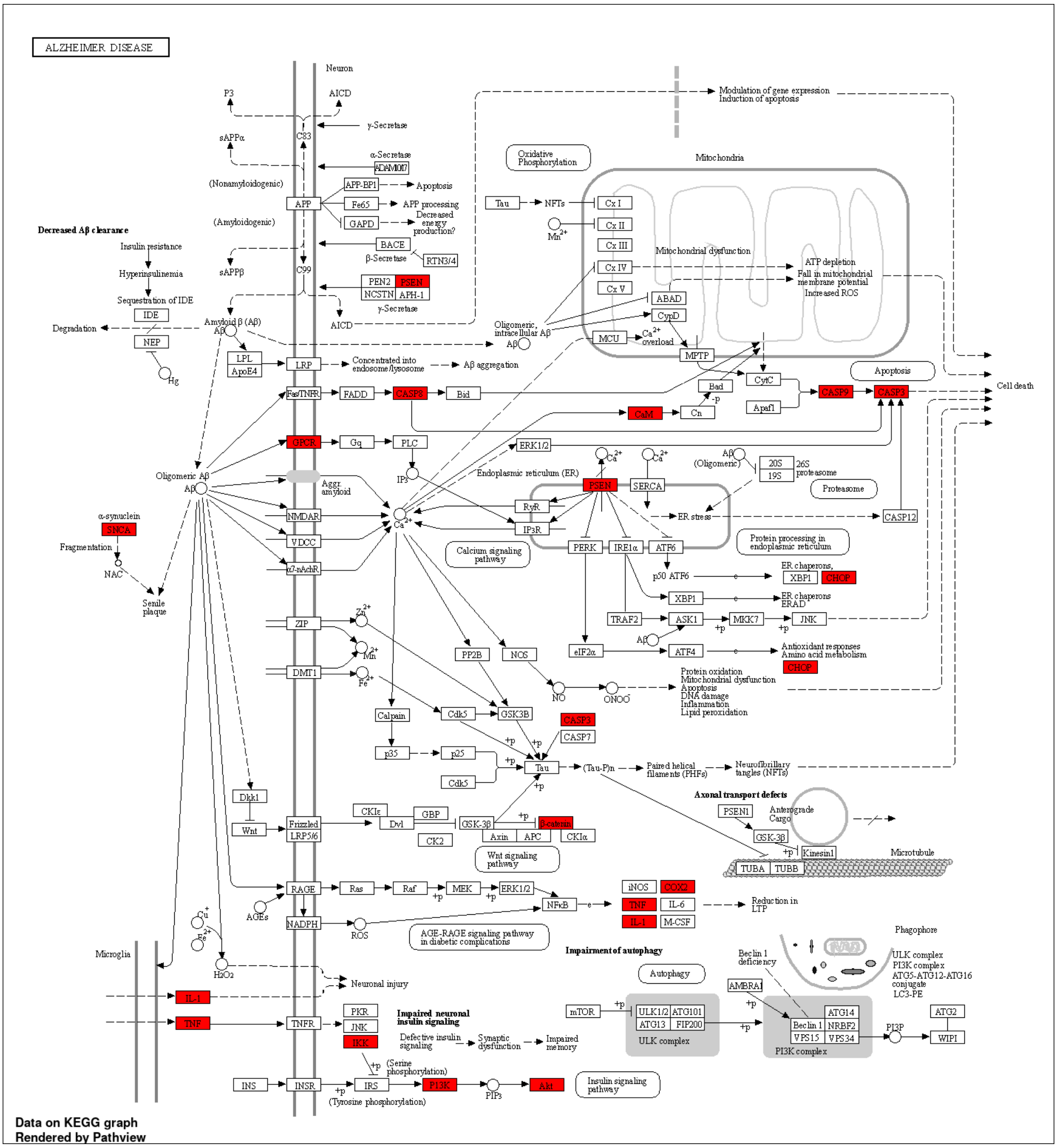

KEGG analysis of I3C with AD. The red-colored proteins are associated with the pharmacological activities of I3C against AD.

Supplementary figure S2: KEGG analysis of DIM

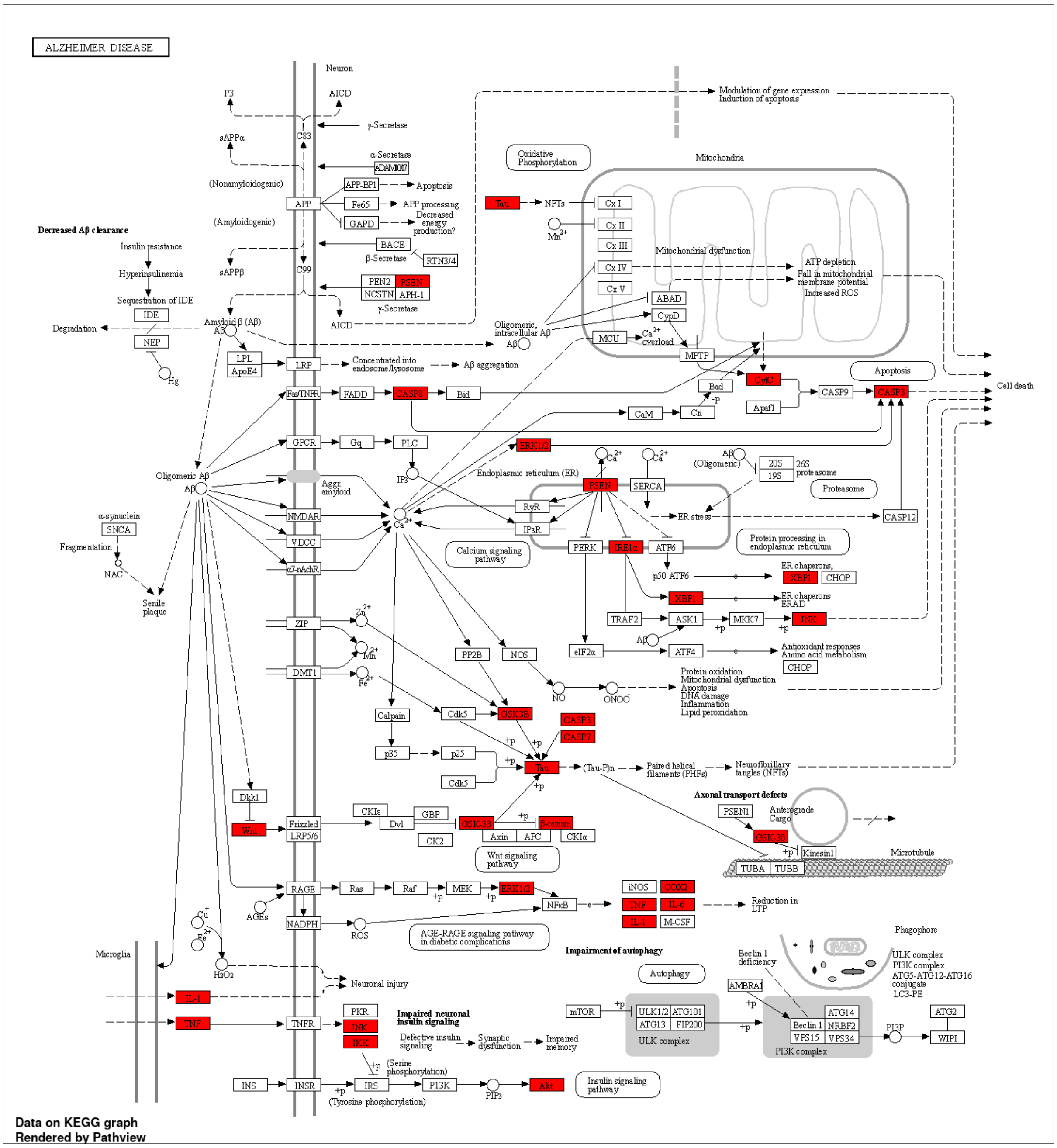

KEGG analysis of DIM with AD. The red-colored proteins are associated with the pharmacological activities of DIM against AD.
